# Supplementary material for: Immune biomarkers, profiles, and responses: a vaccine ontology perspective
Source: J Biomed Semantics. 2026 Jun 27;17:20. doi: 10.1186/s13326-026-00360-x (PMC13602549; doi:10.1186/s13326-026-00360-x)
Supplement: Supplementary file 1 [file 13326_2026_360_MOESM1_ESM.docx]

**Supplemental Table 1. Immune-related issue trackers from biomedical ontologies including the Ontology for Biomedical Investigations (OBI), Ontology of Precision Medicine and Investigation (OPMI), and Vaccine Ontology (VO).**

| **Ontology Issue tracker ID** | **Issue Title** | **Web link** |
| --- | --- | --- |
| OBI #1866  & VO #631 | immunization in both VO and OBI | <https://github.com/obi-ontology/obi/issues/1866> &  <https://github.com/vaccineontology/VO/issues/631> |
| OPMI (#16) | Update definition of immune biomarker | <https://github.com/OPMI/opmi/issues/16> |
| VO #603 | Request for "Correlate of Protection" prefix IRI additions | <https://github.com/vaccineontology/VO/issues/603> |
| VO #840 | VO Immune Marker Refactorization | <https://github.com/vaccineontology/VO/issues/840> |
| VO #845 | New term proposal: 'immune signature' | <https://github.com/vaccineontology/VO/issues/845> |
| VO #846 | Terms related to vaccine antigen processing and presentation | <https://github.com/vaccineontology/VO/issues/846> |
